# Classifying COVID-19 Patients From Chest X-ray Images Using Hybrid Machine Learning Techniques: Development and Evaluation

**DOI:** 10.2196/42324

**Published:** 2023-02-28

**Authors:** Thanakorn Phumkuea, Thakerng Wongsirichot, Kasikrit Damkliang, Asma Navasakulpong

**Affiliations:** 1 College of Digital Science Prince of Songkla University Songkhla Thailand; 2 Division of Computational Science, Faculty of Science Prince of Songkla University Songkhla Thailand; 3 Division of Respiratory and Respiratory Critical Care Medicine Faculty of Medicine Prince of Songkla University Songkhla Thailand

**Keywords:** COVID-19, machine learning, medical informatics, coronavirus, diagnosis, model, detection, healthy, unhealthy, public, usage, data, database, accuracy, development, x-ray, imaging

## Abstract

**Background:**

The COVID-19 pandemic has raised global concern, with moderate to severe cases displaying lung inflammation and respiratory failure. Chest x-ray (CXR) imaging is crucial for diagnosis and is usually interpreted by experienced medical specialists. Machine learning has been applied with acceptable accuracy, but computational efficiency has received less attention.

**Objective:**

We introduced a novel hybrid machine learning model to accurately classify COVID-19, non-COVID-19, and healthy patients from CXR images with reduced computational time and promising results. Our proposed model was thoroughly evaluated and compared with existing models.

**Methods:**

A retrospective study was conducted to analyze 5 public data sets containing 4200 CXR images using machine learning techniques including decision trees, support vector machines, and neural networks. The images were preprocessed to undergo image segmentation, enhancement, and feature extraction. The best performing machine learning technique was selected and combined into a multilayer hybrid classification model for COVID-19 (MLHC-COVID-19). The model consisted of 2 layers. The first layer was designed to differentiate healthy individuals from infected patients, while the second layer aimed to classify COVID-19 and non-COVID-19 patients.

**Results:**

The MLHC-COVID-19 model was trained and evaluated on unseen COVID-19 CXR images, achieving reasonably high accuracy and F measures of 0.962 and 0.962, respectively. These results show the effectiveness of the MLHC-COVID-19 in classifying COVID-19 CXR images, with improved accuracy and a reduction in interpretation time. The model was also embedded into a web-based MLHC-COVID-19 computer-aided diagnosis system, which was made publicly available.

**Conclusions:**

The study found that the MLHC-COVID-19 model effectively differentiated CXR images of COVID-19 patients from those of healthy and non-COVID-19 individuals. It outperformed other state-of-the-art deep learning techniques and showed promising results. These results suggest that the MLHC-COVID-19 model could have been instrumental in early detection and diagnosis of COVID-19 patients, thus playing a significant role in controlling and managing the pandemic. Although the pandemic has slowed down, this model can be adapted and utilized for future similar situations. The model was also integrated into a publicly accessible web-based computer-aided diagnosis system.

## Introduction

COVID-19 has become a widespread pandemic causing high levels of infection and mortality. The first cases were reported in December 2019 and rapidly spread worldwide, leading to its declaration as a severe disease by the World Health Organization (WHO) in May 2020 [1]. Recently, more than 190 million confirmed cases have been reported, with 4 million fatalities worldwide [2]. The virus responsible for COVID-19, a severe acute respiratory syndrome (SARS), was formally named SARS-CoV-2 by the International Committee on Taxonomy of Viruses [3]. An initial study showed that the virus originated from bats and was transmitted to humans by unknown intermediate animals [4,5]. COVID-19 has been divided into 5 clinical stages based on its characteristics: asymptomatic, mild clinical symptoms, moderate clinical features, severe symptoms, and critical cases [6]. The majority of COVID-19 patients have fever, fatigue, cough, shortness of breath, myalgia, and dyspnea [7,8]. However, patients may have asymptomatic COVID-19 disease [9]. Reverse transcription polymerase chain reaction (RT-PCR) performed on throat swab samples is the gold standard for COVID-19 diagnosis [10]. However, RT-PCR results require a considerable amount of time to become available [11]. Rapid diagnostic methods (such as the rapid antigen and antibody tests) are available; however, they cannot substitute for RT-PCR [12-14]. Generally, a chest x-ray (CXR) is prescribed for a high-risk patient—old age, high blood pressure, and chronic respiratory disease—who may be classified as a patient under inspection [15]. Thereafter, a CXR image is examined by medical doctors or specialists for lung infection. A challenging situation occurs because the lung infection may be due not only to SARS-CoV-2 but also to other viruses and bacteria [16]. During the widespread COVID-19 pandemic, many areas with a high level of infection experienced a shortage of physicians.

Recent work explored deep learning and medical imaging techniques to diagnose CXR film of COVID-19 for early detection purposes [17-20]. In [21], the researchers presented a series of steps for classifying COVID-19 and other lung diseases based on 2 data sets: a CXR data set of 1926 images and a computed tomography (CT) scan data set of 2482 images. The results from the CXR data set have an accuracy of 0.993 and an *F*_1_-score of 0.931. Furthermore, the results from the CT scan data set have an accuracy of 0.932 and an *F*_1_-score of 0.921. The experiment used Raspberry Pi Linux and Python code to perform a sequential feature selector. A similar work, documented in [22], implemented VGG16 and Xception to distinguish COVID-19 infections from noninfected cases. They developed 2 models using 1037 CXR images (402 COVID-19 images, 400 normal images, 200 pneumonia images, and 35 images without COVID-19 or pneumonia infection). Regarding this study, each convolutional neural network (CNN) architecture was subjected to 10 rounds of experimentation for model evaluation purposes. The highest accuracy rate achieved was 0.970 for the VGG16 and 0.984 for Xception. An Xception model [23] was proposed to classify COVID-19 CXR images, with 0.896 and 0.950 accuracies for 4 and 3 classes of classification, respectively. The data set consisted of 284 COVID-19, 327 viral pneumonia, 330 bacterial pneumonia, and 310 normal CXR images. In [24], researchers proposed a classification model to classify COVID-19 from CXR images. The study comprised 4290 pneumonia, 1583 normal, and 76 COVID-19 CXR images. Furthermore, pneumonia and normal classes were partially used and split to balance the data set. The researchers focused on the use of a data preprocessing step. An image augmentation technique was used with COVID-19 CXR images. The fine-tuning of a pretrained model, SqueezNet, used Bayesian optimization. The proposed model obtained a test accuracy and *F*-measure of 0.983 and 0.983, respectively. Four transfer learning techniques, ResNet18, ResNet50, SqueezeNet, and DenseNet-121, were used to identify COVID-19 on CXR images in [25]. The COVID-19 CXR data set was divided into 2084 training and 3100 test images. The results showed that SqueezeNet achieved 0.929 specificity and 0.98 sensitivity. However, there was no presence of accuracy of the selected transfer learning techniques. A similar study was conducted in [26]. Pretrained AlexNet, GoogLeNet, and SqueezeNet models were fine-tuned to classify lung infections from CXR images. They used 6 CXR image data sets that were collected from several public databases. The classification results of the pretrained models were promising. Another research work [27] used the transfer learning technique with GoogLeNet, ResNet-18, and DenseNet-12 to perform binary classification of normal and pneumonia images from 2 publicly available CXR image data sets; a 5-fold cross-validation technique was used to evaluate the models. The experimental results showed accuracy and sensitivity of 0.988 and 0.988, respectively, for the first data set and 0.869 and 0.870, respectively, for the second data set. In [28], a CNN-based architecture was proposed to delineate CXR images into 3 categories: healthy, pneumonia, and COVID-19. The data sets were collected from 6 public databases, including 10,451 healthy, 573 COVID-19, and 11,673 pneumonia images. The proposed model achieved an accuracy of 0.912 in the prediction of the 3 classes (healthy, pneumonia, and COVID-19) and an accuracy of 0.982 in the prediction of the 2 classes (COVID-19 or pneumonia). In [29], a new end-to-end trained CNN model was proposed with deep feature extraction and fine-tuning of a pretrained CNN. The proposed model consisted of 3 deep learning approaches for COVID-19 detection. The data set used in this study contained 180 COVID-19 and 200 healthy CXR images. The selected features extracted from the ResNet50 model and support vector machine (SVM) classifiers outperformed other approaches, with a classification accuracy of 0.947. In [30], 5 different pretrained deep models (decision tree [DT], random forest, AdaBoost, bagging, and SVM) were used to classify COVID-19 CXR. With this experiment, the data set contained 1102 CXR images: 565 normal and 537 COVID-19. The data set was divided into training and test sets at a ratio of 70:30. The results showed that the Xception combined with the SVM classifier achieved the best classification result, with accuracy, sensitivity, and specificity of 0.993, 0.992, and 0.993, respectively. This study was exceptional by proposing combinations of traditional machine learning and deep learning techniques. A hybrid ensemble model using MobileNet and Inception V3 was proposed in [31]; 4-fold cross-validation was performed to evaluate the model using a data set of 1050 normal, 1050 viral pneumonia, 1050 bacterial pneumonia, and 1050 COVID-19 CXR images. The classification results for diagnosing COVID-19 from CXR achieved accuracy, precision, and specificity of 0.942, 0.899, and 0.883, respectively. Table 1 shows a summary of related studies on COVID-19 CXR images.

**Table 1:** Related studies of COVID-19 chest x-ray images.

Method	Data set	Balanced data set	Classes, n	Cross-validation	Reference
Sequential feature selection	COVID-19=212, non-COVID-19=1696	No	2	5-fold	[21]
Xception and VGG16	COVID-19=402, pneumonia=200, normal=400, without COVID-19 or pneumonia=35	No	2	No	[22]
CoroNet (Xception)	COVID-19=284, bacterial pneumonia=330, viral pneumonia=327, normal=310	No	3 and 4	4-fold	[23]
Bayes-SqueezeNet	COVID-19=1979, pneumonia=3895, normal=3111	No	3	No	[24]
Different pretrained CNN^a^ model	COVID-19=184, normal=5000	No	2	5-fold	[25]
Different pretrained CNN model	6 different COVID-19 data sets	No	2 and 3	No	[26]
GoogLeNet, ResNet-18, and DenseNet-121	2 different pneumonia data sets	No	2	5-fold	[27]
Customized CNN model	COVID-19=573, normal=10,546, pneumonia=11,673	No	2	No	[28]
ResNet50 for Features extraction + SVM^b^	COVID-19=180, normal=200	No	2	No	[29]
Xception + SVM	COVID-19=537, normal=565	No	2	10-fold	[30]
Hybrid ensemble model	COVID-19=1050, viral pneumonia=1050, bacterial pneumonia=1050, normal=1050	Yes	4	4-fold	[31]
MLHC-COVID-19	COVID-19=1050, non-COVID-19=2100, healthy=1050	Yes	3	10-fold	Our proposed method

^a^CNN: convolutional neural network.

^b^SVM: support vector machine.

According to the literature review, most of the reported studies used deep learning and a pretrained model with data augmentation for COVID-19 detection from CXR images. However, there are several limitations that remain to be addressed. These limitations include (1) the use of an imbalanced data set, (2) the use of deep learning techniques that require significant computational resources, and (3) longer time consumption of the training model. The main contributions of our study, with the aim of addressing these issues, are as follows:

1. We proposed a multilayer hybrid classification model for COVID-19 (MLHC-COVID-19) detection. Our model integrates several machine learning techniques applied to a large CXR image data set of 3 different categories: healthy, non-COVID-19 (viral and bacterial pneumonia), and COVID-19. The MLHC-COVID-19 model was evaluated through a 10-fold cross-validation process to assess its classification performance.
2. The MLHC-COVID-19 is composed of 2 layers of binary classification. The first layer acted as a screening mechanism, directing unhealthy CXR images to the second layer for further classification into COVID-19 and non-COVID-19 images. This model has been thoroughly compared with other preprocessing techniques and methods to assess its effectiveness.
3. The first layer of the MLHC-COVID-19 uses the highest performance model between DTs, SVMs, and neural networks (NNs) to differentiate between healthy and unhealthy CXR images. The second layer uses the most effective model between the same 3 techniques to distinguish between COVID-19 and non-COVID-19 CXR images. The model performance was evaluated through 10-fold cross-validation.
4. We developed a web-based application for the MLHC-COVID-19 model, allowing for practical use in diagnosing COVID-19 from CXR images. The web-based application can be accessed at [32].

This paper is organized as follows: The proposed method (MLHC-COVID-19) is discussed in the Methods section. The Results section presents the in-depth experimental results, compared with deep learning–based methods. The conclusion of the study and suggestions for future research are discussed in the Discussion section.

## Methods

### Data Set

**Figure 1:**
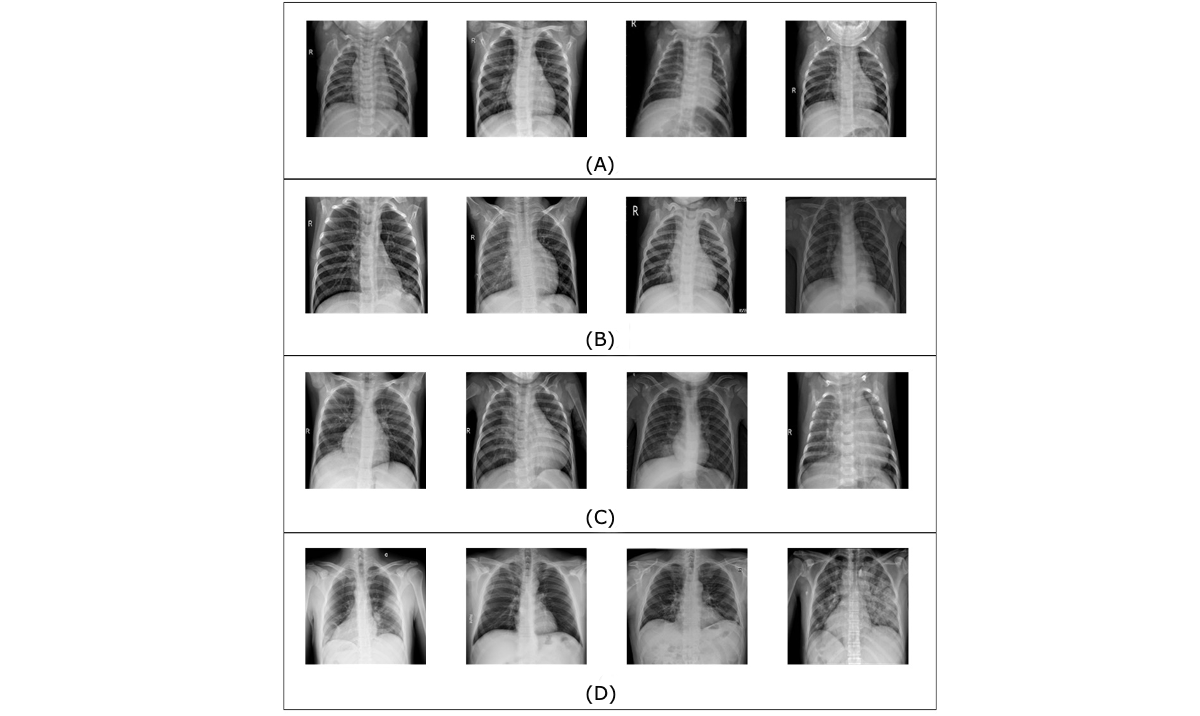
Samples of the chest x-ray images in the combined data set: (A) heathy images, (B) viral pneumonia images, (C) bacterial pneumonia images, (D) COVID-19 images.

In a real-world situation, lung infection can be due to many factors. The distinction of our work lies in the combination of various types of lung infection and healthy CXR images from different data sources. The target classes were healthy, COVID-19, viral pneumonia, and bacterial pneumonia. We used 5 data sets of CXR images that were made publicly available by Mendeley [33], the Italian Society of Medical and Interventional Radiology [34], GitHub [35], Radiopaedia [36], and Kaggle [37]. The total number of images used in this study was 4200. We utilized an annotated data set of CXR images consisting of 3 distinct classes: (1) healthy individuals, (2) individuals infected with non-COVID-19 diseases (viral and bacterial pneumonia), and (3) individuals infected with COVID-19. The COVID-19 class was comprised of 1050 CXR images, of which 912 images were obtained from the first 4 sources and 138 images were obtained from the last source. The non-COVID-19 and healthy classes were comprised of a total of 3150 CXR images, with 1050 images being healthy and 2100 images being non-COVID-19. The annotation of each image was performed by skilled medical specialists. Table 2 shows a summary of the CXR images data set used in this study, and samples of the CXR images in the combined data set are shown in Figure 1.

**Table 2:** Chest x-ray image data set.

Open source/classes		Number of images, n
**Kaggle [37]**		
	Healthy	1050
	Non-COVID-19	2100
**Mendeley data set [33]**		
	COVID-19	912
**SIRM^a^ [34], GitHub [35], and Radiopaedia [36]**		
	COVID-19	138

^a^SIRM: Italian Society of Medical and Interventional Radiology.

### Overview

**Figure 2:**
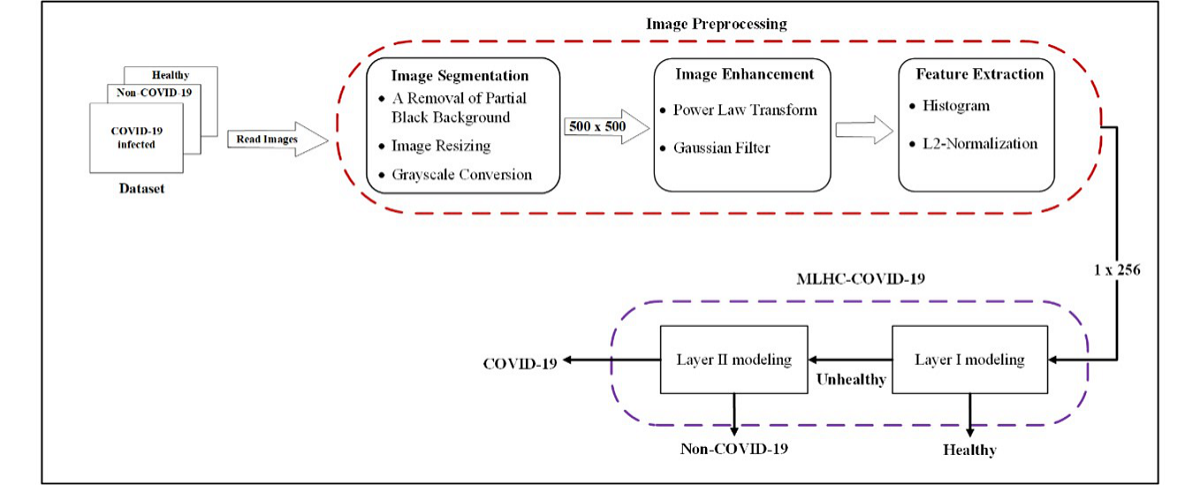
Block diagram of the proposed multilayer hybrid classification model (MLHC)-COVID-19.

The general overview of the proposed MLHC-COVID-19 for identifying CXR images of COVID-19 infection is shown in Figure 2. The block diagram depicts vital processes that were embedded in the model. The following subsections will discuss each of the processes.

### Image Preprocessing

**Figure 3:**
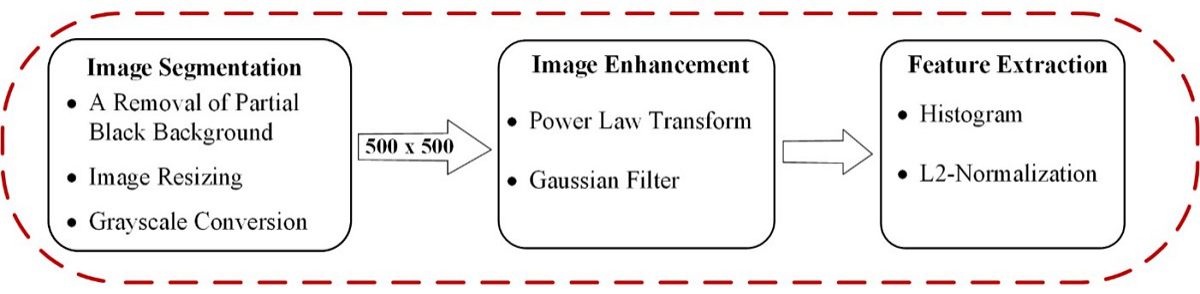
Image preprocessing.

**Figure 4:**
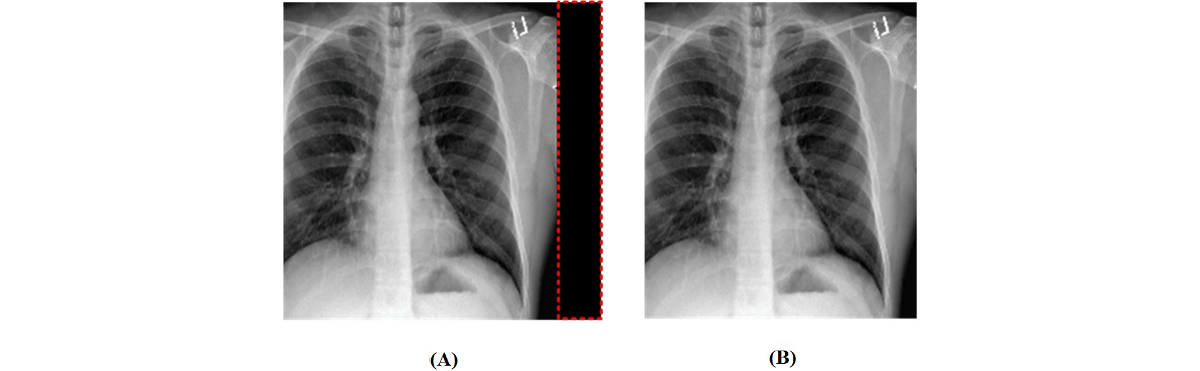
(A) An original chest x-ray (CXR) image with a partial black background and (B) the CXR image after the removal of the partial black background.

In this study, image preprocessing consisted of 3 steps (Figure 3): image segmentation, image analysis, and feature extraction. Considering the image segmentation, we designed 3 substeps: removal of the partial black background, image resizing, and grayscale conversion. The combined data set had different formats and a partial black background in the CXR images. This was attributed to several factors including the positioning during the filming process and the type of x-ray machines utilized. The black background affects the classification performance. We programmatically removed the black background from the CXR image. Figure 4 shows an example of a completed programmatic removal of a black background. Furthermore, the CXR images were of different dimensions; therefore, all the images were resized to an identical dimension of 500 × 500 pixels. The last step of the image segmentation was the conversion of the images to grayscale to reduce the image features. Reduction of the image features is known to improve the classification result and mimic the complexity of the algorithm [38]. The grayscale formula is given by equation 1.

G_intensity_(x,y)=0.2989 × f(x,y,R) + 0.5871 × f(x,y,G) + 0.1140 × f(x,y,B) **(1)**

where, if *G_intensity_*(*x*,*y*) is an image with grayscale, then *f*(*x*,*y*,*R*) is a pixel value in the (*x*,*y*) coordinates of the red channel, *f*(*x*,*y*,*G*) is a pixel value in the (*x*,*y*) coordinates of the green channel, and *f*(*x*,*y*,*B*) is a pixel value in the (*x*,*y*) coordinates of the blue channel [39].

**Figure 5:**
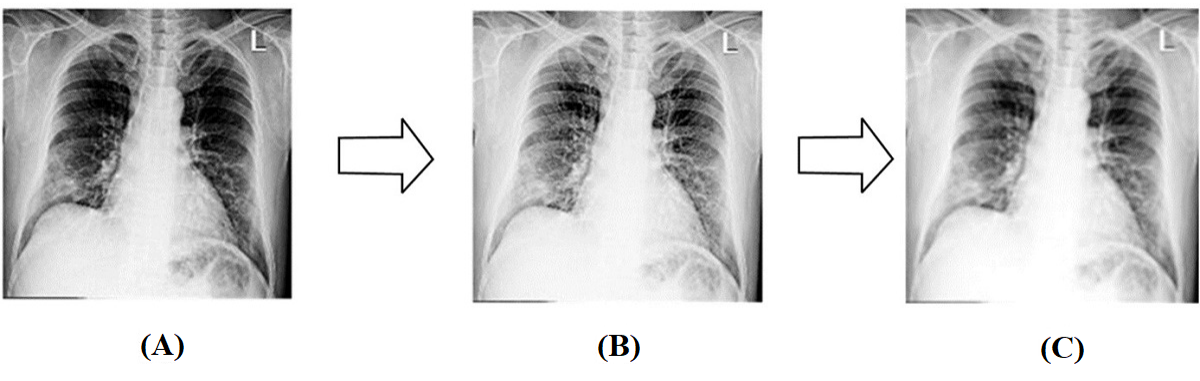
Image after the (A) image segmentation, (B) power law transformation, (C) Gaussian filter.

The second step in the image preprocessing was image enhancement. We utilized the power law transformation to adjust the brightness of the CXR images. We applied a *γ* value of 0.5. The power-law transformation formula is shown in equation 2 [40,41]. In addition, we selected the 2-dimensional Gaussian filter technique to reduce the Gaussian and salt-and-pepper noises [42]. The Gaussian filter technique is given by equation 3. Figure 5 shows the processed CXR image after the image enhancement.

s=c × r^γ^
**(2)**

where *s* is the output pixel value, *c* is a value of the normalized image, *γ* is the gamma value, and *r* is the input pixel value.



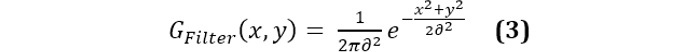



where *∂*^2^ is the variance of the Gaussian filter with 3 x 3 kernel size and *x* and *y* are the horizontal and vertical axes, respectively, of the kernel size [43].

**Figure 6:**
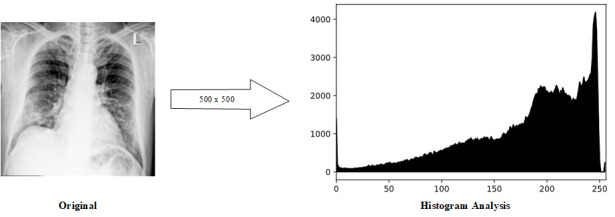
The final output of the image preprocessing step.

The last step in image preprocessing is feature extraction. We performed histogram analysis and L2-normalization. Histogram analysis reduces the image features by retrieving vital image statistics. Specifically, low-intensity values dictate an image in a dark tone and vice versa [44]. Upon completion of the histogram analysis, each feature in the histogram has different scales. Subsequently, to achieve the same standard scale, we performed L2-normalization. L2-normalization or the Euclidean norm normalizes the features of the histogram into the same scale using equation 4. Figure 6 shows the output of feature extraction.



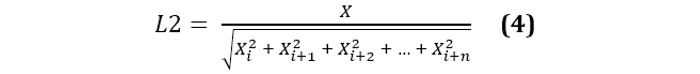



where *X* is a feature of the histogram.

### Multilayer Hybrid Classification Model

First, the original MLHC was developed to automatically classify the multisleep stages. Each layer in the MLHC is a binary classification model that uses different machine learning techniques [45]. The MLHC model was a stacking-based machine learning model that utilized multiple models to improve its performance. “Multilayer” refers to the stacking of multiple models, while “hybrid” signifies the combination of different model types, such as DTs, SVMs, and NNs. The primary objective of the MLHC was to diagnose COVID-19 from CXR images. By stacking various models, the MLHC aimed to take advantage of the strengths of each model and enhance the accuracy and robustness of the system in diagnosing COVID-19 patients.

**Figure 7:**
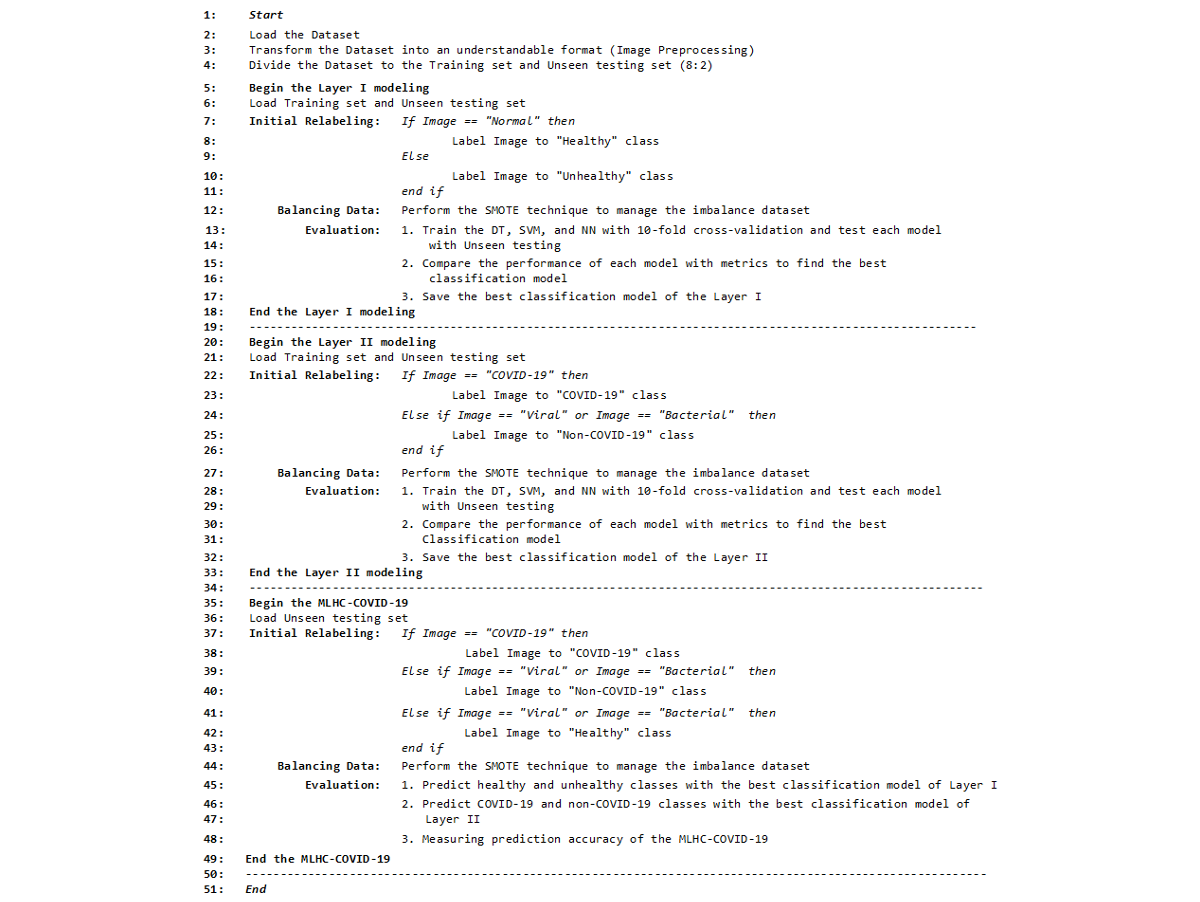
Pseudocode of the multilayer hybrid classification model (MLHC)-COVID-19.

**Figure 8:**
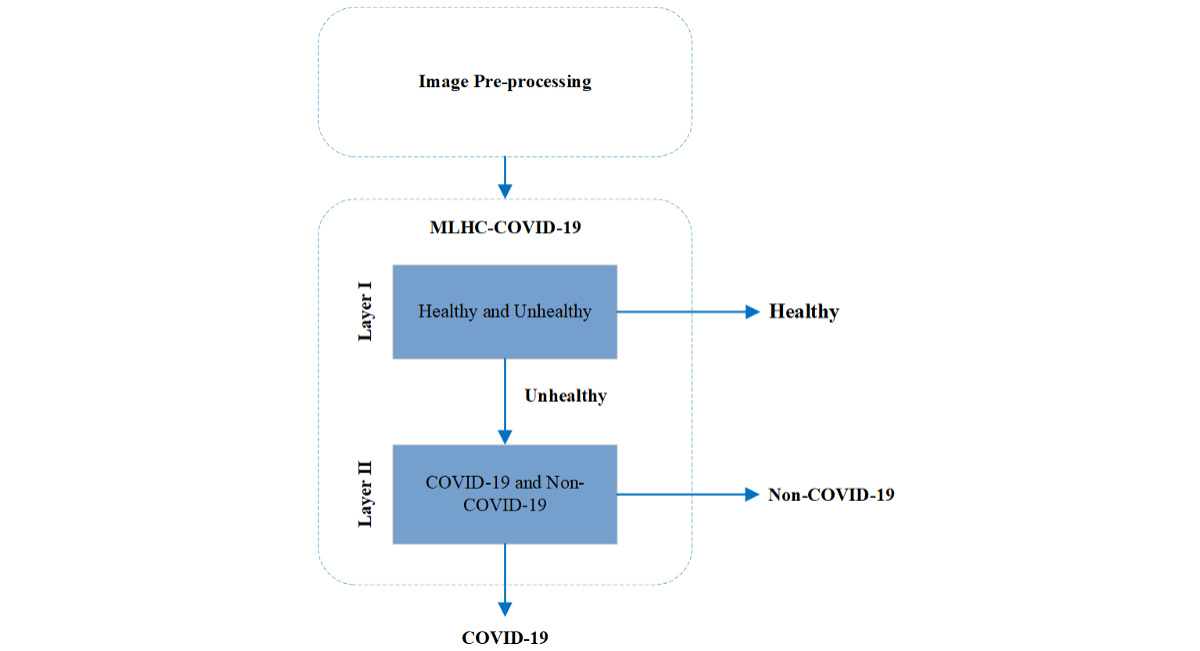
Flowchart of multilayer hybrid classification model (MLHC)-COVID-19.

The MLHC-COVID-19 model in the experiment was designed using a 2-step approach. The first step aimed to differentiate between healthy and unhealthy individuals, where the latter included those infected with viral, bacterial, or COVID-19 infection. Once the differentiation was made, the infected individuals were then directed to the second step, which was to classify them further into either COVID-19 or non-COVID-19 cases (consisting of viral and bacterial infections). Figures 7 and 8 show the MLHC-COVID-19.

Regarding our experiment, we evaluated 3 machine learning techniques: DT, SVM, and NN. These selected techniques were candidates for embedding into MLHC-COVID19. DT is a well-known supervised machine learning method. Each node represents a condition for decision on data classification, in which various branches of trees represent results from testing and the leaves of the DT represent the classification [46,47]. DT is one of the simplest techniques to understand and is suitable for classification tasks [48]. SVM is a supervised machine learning method. It exhibits promising performance in statistical classifications [49]. It distinguishes data by finding hyperplanes as separators. The process of identifying hyperplanes is iteratively toward the best line during the training [50]. We selected the radial basis function as the kernel function [51]. Finally, NNs are types of mathematical models for processing data with connected computation nodes that mimic the functions of biological NNs [52]. It builds complex models between the inputs and outputs with high efficiency [53]. We designed 4 fully connected layers (dense layers) and 2 dropout layers. The input to the first dense layer consisted of 256 histograms. The first and second dense layers used 128 neurons, and the third dense layer used 32 neurons. The last dense layer was fed into the softmax classifier [54]. In addition, the dropout layers, with a ratio of 0.2, intervened between the dense layers [55].

### Performance Evaluation

We divided the data set into a training set and a testing set at a ratio of 80:20. The training set was utilized to train the MLHC-COVID-19 model, and its performance was evaluated through 10-fold cross-validation. The testing set, representing unseen data, was utilized to select the optimal model among the models generated in the training phase. The performance measures were accuracy, sensitivity, specificity, precision, *F* measure, and area under the curve (AUC) [56,57]. The performance measure formulae are given in equations 5-10, and the measured performances included the true positive (TP), true negative (TN), false positive (FP), and false negative (FN). In addition, the classification performance was evaluated using the area under the receiver operating characteristic curve (AUC of the ROC). We used the Python module 3.8.3 known as Scikit-learn for machine learning algorithms [58] with an NVIDIA GeForce-960 M GPU, 4 GB GDDR5 onboard memory with Intel Core i7-6700 HQ (2.60 GHz, 6 MB L3 cache, approximately 3.50 GHz), 8 GB DDR4 RAM, and 1 TB hard drive.



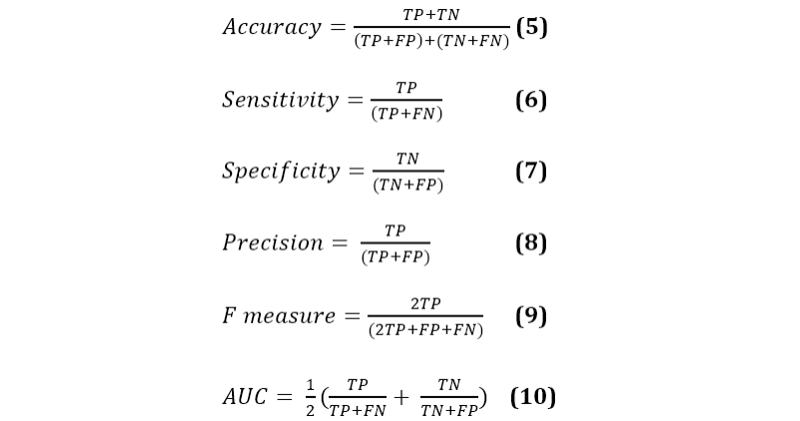



## Results

Considering the MLHC-COVID-19 design, the distribution of data in each layer was imbalanced, potentially causing bias in the evaluation. To solve the imbalance issue, we used the synthetic minority oversampling technique (SMOTE) to balance the classes [59]. The SMOTE synthesized new data from the existing data using k-nearest neighbors and inserted them into the original data set [60]. The results of using SMOTE are presented in Table 3, which shows the number of images in each class of the original and the SMOTE-augmented data sets based on the MLHC-COVID-19 design. For instance, the original data set consisted of 840 images in the healthy class and 2520 images in the unhealthy class, resulting in the healthy class being the minority class in Layer I. To balance both classes, 1680 synthetic healthy instances were generated using SMOTE.

We investigated 3 machine learning techniques: DT, SVM, and NNs. The best classification model for each layer was selected for the MLHC-COVID-19. Table 4 shows the classification results for training and testing. During training, 10-fold cross-validation was performed, and the results are shown as the average (SD). First, the NNs achieved the highest accuracy at 0.983 (SD 0.013) during the training, followed by SVM and DT in Layer I. In addition, the NNs achieved the highest position with a mean sensitivity of 0.987 (SD 0.012), specificity of 0.980 (SD 0.022), precision of 0.979 (SD 0.023), *F* measure of 0.983 (SD 0.013), and AUC of 0.995 (SD 0.005). Considering the time, the DT minimized the time spent in Layer I. Considering Layer II, the SVM had the highest performance metric in all the aspects during the training (mean AUC 0.984, SD 0.004). The second best was the NN, followed by the DT.

**Table 3:** Comparison of the number of instances of the original and applied synthetic minority oversampling technique (SMOTE) data sets.

MLHC-COVID-19^a^ class		Images in the training set (80%), n	
		Original	SMOTE
**Layer I**			
	Healthy	840	2520
	Unhealthy	2520	2520
**Layer II**			
	COVID-19	840	1680
	Non-COVID-19	1680	1680

^a^MLHC: multilayer hybrid classification model for COVID-19.

**Table 4:** 10-fold cross-validation results.

Layer and performance metric		Train (10-fold cross-validation)		
		DT^a^, mean (SD)	SVM^b^, mean (SD)	NN^c^, mean (SD)
**Layer I (healthy, unhealthy)**				
	Accuracy	0.863 (0.018)	0.962 (0.006)	0.988 (0.010)^d^
	Sensitivity	0.719 (0.048)	0.972 (0.009)	0.992 (0.007)^d^
	Specificity	0.912 (0.009)	0.953 (0.012)	0.985 (0.015)^d^
	Precision	0.728 (0.042)	0.954 (0.011)	0.985 (0.014)^d^
	*F* measure	0.723 (0.040)	0.963 (0.005)	0.989 (0.009)^d^
	AUC^e^	0.819 (0.025)	0.962 (0.006)	0.995 (0.005)^d^
	Training time (sec)	1.108 (0.066)^d^	1.201 (0.123)	24.888 (1.277)
**Layer II (COVID-19, non-COVID-19)**				
	Accuracy	0.936 (0.012)	0.985 (0.004)^d^	0.979 (0.005)
	Sensitivity	0.950 (0.012)	0.981 (0.007)^d^	0.977 (0.007)
	Specificity	0.908 (0.026)	0.987 (0.005)^d^	0.981 (0.015)
	Precision	0.954 (0.011)	0.987 (0.005)^d^	0.981 (0.015)
	*F* measure	0.952 (0.009)	0.984 (0.004)^d^	0.979 (0.005)
	AUC	0.929 (0.015)	0.984 (0.004)^d^	0.979 (0.005)
	Training time (sec)	0.749 (0.070)	0.275 (0.020)^d^	18.183 (0.493)

^a^DT: decision tree.

^b^SVM: support vector machine.

^c^NN: neural network.

^d^Best classification results.

^e^AUC: area under the curve.

**Figure 9:**
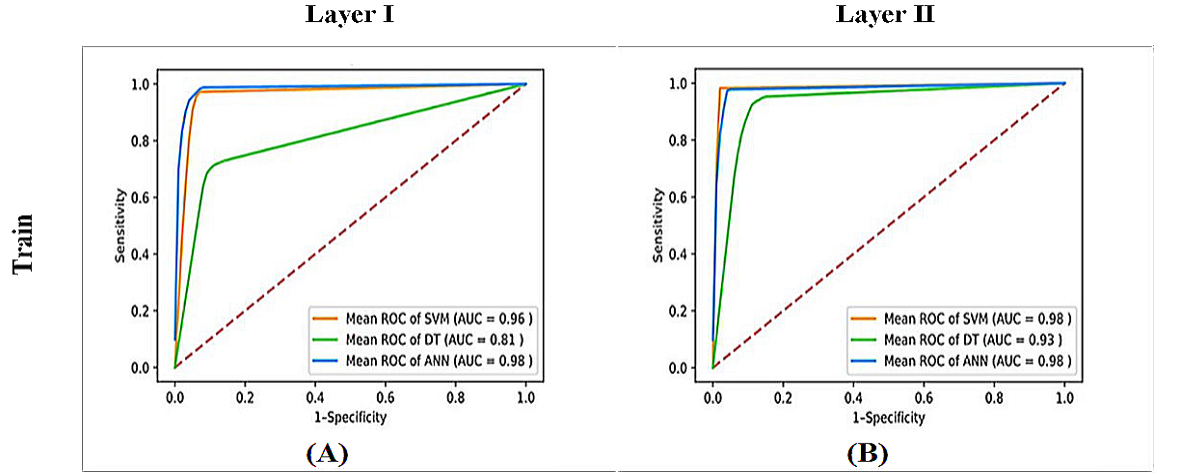
Receiver operating characteristic (ROC) in (A) Layer I and (B) Layer II. ANN: artificial neural network; AUC: area under the curve; DT: decision tree; SVM: support vector machine.

Figure 9 shows a graphical representation of the averaged ROC of the 0-fold cross-validation of the training. The NNs achieved the best classification result in Layer I, and SVM outperformed all the other models in Layer II.

Considering the 10-fold cross-validation, the proposed MLHC-COVID-19 model consisted of 2 layers, in which NNs were utilized in the first layer and SVM was utilized in the second layer. The first layer was designed to differentiate between healthy and unhealthy based on CXR images. The second layer then classified the infected individuals into either COVID-19 or non-COVID-19. For the testing, we separated 20% of the data set as mentioned in the previous section. The classification results in the unseen test also confirmed that the NNs and SVM are the best classifiers in Layers I and II, respectively. However, to address the issue of class imbalance in the unseen test set, we applied the SMOTE to the data as shown in Table 5.

**Figure 10:**
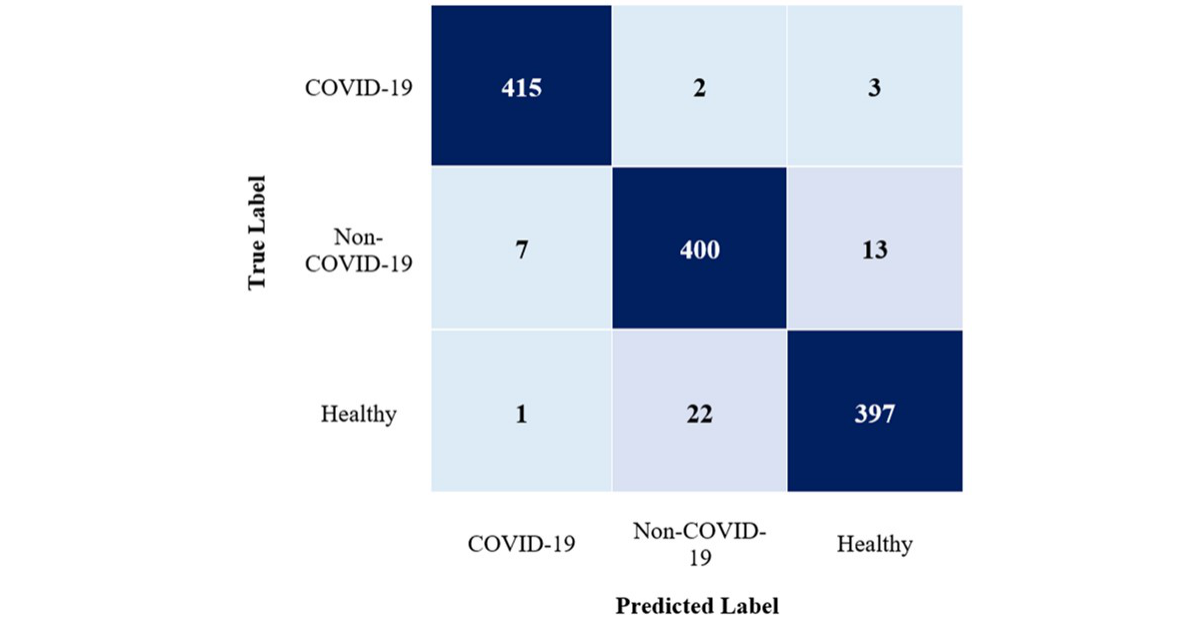
Confusion matrix of the multilayer hybrid classification model (MLHC)-COVID-19.

The MLHC-COVID-19 model demonstrated superior performance compared with other techniques in the unseen test results. As presented in Table 6, the accuracy and *F* measure of the MLHC-COVID-19 model in the unseen test set were 0.962 and 0.962, respectively. Figure 10 displays the full confusion matrix of the MLHC-COVID-19 in the unseen test. The MLHC-COVID-19 correctly identified 415 cases of COVID-19. There were 2 COVID-19 cases that were misclassified as non-COVID-19, and 3 COVID-19 cases were incorrectly classified. Conversely, there were 8 cases (7 non-COVID-19 and 1 healthy) that were incorrectly classified as COVID-19 cases. For the non-COVID-19 class, 7 cases were misclassified as COVID-19, while 400 cases were correctly classified, and 13 cases were misclassified as healthy. For the healthy class, only 1 case was misclassified as COVID-19, 22 cases were misclassified as non-COVID-19, and 397 cases were correctly classified.

**Table 5:** Results of synthetic minority oversampling technique (SMOTE) on the unseen testing set.

Class	Images in the testing set (20%), n	
	Original	SMOTE
COVID-19	210	420
Non-COVID-19	420	420
Healthy	210	420

**Table 6:** Classification results from the multilayer hybrid classification model (MLHC)-COVID-19.

Class	Precision	Sensitivity	*F* measure	Accuracy
COVID-19	0.981	0.988	0.985	—^a^
Non-COVID-19	0.943	0.952	0.948	—
Healthy	0.961	0.945	0.953	—
Macro-average	0.962	0.962	0.962	0.962

^a^Not assessed.

## Discussion

### Principal Findings

Our study showed that the MLHC-COVID-19 model achieved promising results in terms of accuracy, sensitivity, and precision for classifying healthy, non-COVID-19, and COVID-19 images. Image preprocessing played a crucial role in obtaining meaningful information and accurate classification by eliminating noisy or distorted pixels from each image. This allowed the classification model to diagnose infections from CXR images effectively. Additionally, histogram analysis of CXR images and L2-normalization reduced the training time of the model to less than 30 seconds. The data set was also divided based on a newly proposed multilayer design.

### Comparison With Prior Work

Diagnosis of COVID-19 was based on analyzing or classifying CXR images. Recent studies showed various preprocessing techniques, feature extraction methods, and classification approaches. Two main deep learning techniques, the customized CNN and ensemble learning methods, were extensively used in most recent research. Due to the complexity of the deep learning techniques, they require extensive computer processing performance. We aimed to develop the MLHC-COVID-19 model to reduce the processing time compared with other deep learning techniques while still maintaining acceptable performance. Our objective was to achieve a reliable COVID-19 diagnosis from CXR images and reduce the likelihood of misdiagnosis. The model achieved an accuracy of over 95%, which was higher than other similar work. These results are detailed in Table 7, which compares our model with those in other studies. Our MLHC-COVID-19 model outperformed the hybrid ensemble model, which achieved an accuracy of 0.942, sensitivity of 0.884, precision of 0.899, and *F* measure of 0.886 on the same data set in [31]. Our proposed model achieved a higher accuracy of 0.962 and *F* measure of 0.962.

**Table 7:** Comparison of the COVID-19 classification results.

Number of classes	Hybrid or ensemble model	Accuracy	Sensitivity	Specificity	Precision	*F* measure	AUC^a^	Study
2	No	0.990	0.968	0.991	0.909	0.938	—^b^	[21]
2	Yes	0.969	0.954	0.976	0.954	0.954	—	[22]
3	No	0.950	0.969	0.975	0.950	0.956	—	[23]
3	Yes	0.983	—	0.991	—	0.983	—	[24]
2	Yes	—	0.980	0.900	—	—	—	[25]
3	Yes	0.975	0.954	0.9776	0.985	0.969	0.999	[26]
3	No	0.912	—	—	—	—	—	[28]
2	Yes	0.947	0.911	0.980	0.976	0.943	—	[29]
2	Yes	0.993	0.990	0.994	0.993	0.993	0.993	[30]
4	Yes	0.942	0.884	—	0.899	0.886	—	[31]
3	No	0.962	0.962	—	0.962	0.962	—	MLHC-COVID-19^c^

^a^AUC: area under the curve.

^b^Not assessed.

^c^MLHC: multilayer hybrid classification model for COVID-19.

### Limitations

The MLHC-COVID-19 model has some limitations that can be improved upon. Currently, the model is designed for global classification, meaning it cannot identify specific abnormal regions within CXR images. We plan to explore other data preprocessing techniques beyond histogram analysis that may lead to better classification results. Additionally, there are still other techniques available for COVID-19 classification that we aim to explore. Last, our goal is to enhance the MLHC-COVID-19 model by adding another layer that can classify viral pneumonia and bacterial pneumonia, resulting in a 4-class classification system.

### Conclusion

We developed the MLHC-COVID-19 to diagnose COVID-19 from CXR images. Our model combines multiple individual models to leverage the strengths of each and improve the overall accuracy and robustness of the system for diagnosing COVID-19. The MLHC-COVID-19 model was designed to differentiate COVID-19–infected CXR images from healthy and non-COVID-19 images, such as those affected by viral or bacterial pneumonia. The model was thoroughly evaluated and compared with other preprocessing techniques and methods to assess its effectiveness. The findings of this study were acceptable when compared with the other techniques. Considering the current situation, the computer-aided diagnosis tool must be easily accessible; therefore, a web-based solution is also feasible. In terms of the practical implication, we developed a prototype for a web-based computer-aided diagnosis tool [32] as an alternative to bring the MLHC-COVID-19 technique into the clinical setting as a useful tool for supporting radiologists in improving COVID-19 accuracy. The main system of this tool allows users to upload CXR images, which are then diagnosed by the MLHC-COVID-19. The tool will evaluate whether the image is healthy, non-COVID-19, or COVID-19.

## Abbreviations

AUC: area under the curve
CNN: convolutional neural network
CT: computed tomography
CXR: chest x-ray
DT: decision tree
FN: false negative
FP: false positive
MLHC: multilayer hybrid classification model
NN: neural network
ROC: receiver operator characteristic
RT-PCR: reverse transcription polymerase chain reaction
SARS: severe acute respiratory syndrome
SMOTE: synthetic minority oversampling technique
SVM: support vector machine
TN: true negative
TP: true positive
WHO: World Health Organization

## Data Availability

The data are available at [61].
